# Multidimensional Liquid Chromatography Coupled with Tandem Mass Spectrometry for Identification of Bioactive Fatty Acyl Derivatives

**DOI:** 10.3389/fphys.2016.00608

**Published:** 2016-12-15

**Authors:** Erin B. Divito, Kristin M. Kroniser, Michael Cascio

**Affiliations:** Department of Chemistry and Biochemistry, Duquesne UniversityPittsburgh, PA, USA

**Keywords:** bioactive lipids, fatty acyls, lipid-protein interactions, multidimensional liquid chromatography, N-acyl ethanolamines, N-acyl glycines, primary fatty acid amides

## Abstract

Recognition of the contributions of lipids to cellular physiology, both as structural components of the membrane and as modulatory ligands for membrane proteins, has increased in recent years with the development of the biophysical and biochemical tools to examine these effects. Their modulatory roles in ion channels and transporters function have been extensively characterized, with the molecular mechanisms of these activities being the subject of intense scrutiny. The physiological significance of lipids in biochemistry is expanding as numerous fatty acyls are discovered to possess signaling properties. These bioactive lipids are often found in quantities of pmol/g of tissue and are co-extracted with numerous lipophilic molecules, making their detection and identification challenging. Common analytical methodologies involve chromatographic separation and mass spectrometric techniques; however, a single chromatographic step is typically ineffective due to the complexity of the biological samples. It is, therefore, essential to develop approaches that incorporate multiple dimensions of separation. Described in this manuscript are normal phase and reversed phase separation strategies for lipids that include detection of the bioactive primary fatty acid amides and N-acyl glycines via tandem mass spectrometry. Concerted utilization of these approaches are then used to separate and sensitively identify primary fatty acid amides extracted from homogenized tissue, using mouse brains as a test case.

## Introduction

Lipids are classically defined as hydrophobic or amphiphilic small molecules with limited solubility in aqueous solutions where they typically aggregate non-covalently. The macromolecular complexes are constituents of cellular membranes or comprise relatively inert complexes used for energy storage. In considering the former, the lipid composition of the bilayer alters its physicochemical properties, and this local environment for receptors and other membrane proteins affect their structure and function (Coskun and Simons, 2011; Laganowsky et al., 2014). Given the focus of this thematic issue, if we restrict discussion to receptors, there are numerous examples of how the structure and function of these classes of membrane proteins are affected by lipids (for overview, see Chattopadhyay and Ruysschaert, 2015). Some dramatic examples include the radically different topologies exhibited by lactose permease as a function of phospholipid composition (Serdiuk et al., 2015), the effects of cholesterol on ion channels and other membrane proteins (Levitan et al., 2014; Korinek et al., 2015; Lange and Steck, 2016), the functional modulation of metabotropic serotonin receptors by bilayer composition (Gutierrez et al., 2016), and the lipid dependence of glucose transporters (Hresko et al., 2016). In turn, the activity of these signaling molecules on the local membrane potential affects the nanoscale organization of the neighboring lipids, potentially affecting cellular signaling properties as shown for K-Ras signaling (Zhou et al., 2015).

In addition to these indirect mutual effects of lipids and signaling, many lipids exert bioactive properties directly as cellular signals and 2nd messengers (for reviews, see Hannun and Obeid, 2008; García-Morales et al., 2015; Morales-Lázaro and Rosenbaum, 2015). In this study, we have focused attention on fatty acyls (for review see Divito and Cascio, 2013). N-acyl ethanolamines (NAEs) have a long history of physiological and G-protein coupled receptor mediated effects that are directly related to, or reminiscent of, cannabinoid activation (Lambert et al., 2002; Ahn et al., 2008; Luchicchi and Pistis, 2012; Mechoulam and Parker, 2013). Primary fatty acid amides (PFAMs) and N-acyl glycines (NAGs), however, have been more recently recognized as bioactive signaling lipids that have demonstrated interactions with serotonin receptors (Huidobro-Toro and Harris, 1996; Huidobro-Toro et al., 1996; Thomas et al., 1997, 1999), gap junction proteins and calcium signaling (Guan et al., 1997; Boger et al., 1998; Huang and Jan, 2001; Lo et al., 2001; Rimmerman et al., 2008), and a myriad of physiological effects (Wakamatsu et al., 1990; Lerner et al., 1994; Cravatt et al., 1995; Mitchell et al., 1996; Basile et al., 1999; Fedorova et al., 2001; Huitrón-Reséndiz et al., 2001; Laposky et al., 2001; Mendelson and Basile, 2001; Murillo-Rodríguez et al., 2001; Stewart et al., 2002). Of these documented effects, the most potent bioactive lipids consist of 16–22 chain length acyl tails and can be saturated or unsaturated species. It is hypothesized that aberrant endogenous levels of these species, either by down-stream modulation or alteration of their metabolic enzymes, could contribute to disease states. This is an intriguing consideration as PFAMs have high affinity for serotonin receptors (Huidobro-Toro and Harris, 1996; Huidobro-Toro et al., 1996; Thomas et al., 1997; Lambert et al., 2002), though no reports exist for PFAM levels in a depressive phenotype. Evidence for up-regulation of NAEs exists in studies of chronic pain (Ghafouri et al., 2013), schizophrenia (Leweke et al., 1999), and depression and anxiety (Dlugos et al., 2012), yet the physiological relevance of these observations is still unclear. In light of this, it is of substantial importance to develop analysis strategies to sensitively detect and accurately quantitate bioactive fatty acyls. These methods, in combination with phenotype studies, will aid in the elucidation of the role of signaling lipids in physiology and help define their role or diagnostic value in health and disease. The generated methodologies may provide additional tools for diagnoses and development of treatment strategies.

Lipid milieus of biological samples are often very complex mixtures. Even the most common lipid extraction methods, such as Folch-Pi (Folch et al., 1957), remove a mélange of several different lipid classes. These include fatty acids, monoacylglycerols, diacylglycerols, triacylglycerols, PFAMs, NAGs, and NAEs. Identification of isomers and isobars by standard separation methods, such as normal phase or reversed phase chromatography, is often insufficient for complete identification of all constituents.

Multidimensional liquid chromatography (MDLC) analysis is the process of separating samples with at least, but most typically, two different chromatographic separation schemes (columns or dimensions)(Giddings, 1984; Bushey and Jorgenson, 1990; Dixon et al., 2006; Cohen, 2008; Dugo et al., 2008). The main advantage of MDLC is the dramatic increase in peak capacity known as the “product rule”; where the maximum peak capacity becomes the product of the individual chromatographic peak capacities. These different separation schemes should be orthogonal, or operate by different separation mechanisms, in order to achieve maximum discrimination and capacity. Analyses by two or more different chromatographic dimensions allows for the separation of difficult to resolve components, or samples with a high number of constituents. The wide and complex lipid composition in biological samples require MDLC methodologies to sufficiently separate the lipids prior to MS or MS/MS characterization (as an additional complication may arise due to isobaric lipids). Lipid separations have been demonstrated with normal phase, reversed phase and silver ion chromatography.

Fortuitously, straight chain fatty acyls have a well-predicted elution order by reversed phase chromatography where retention is directly proportional to the length of the acyl chain. The degrees of unsaturation reduce the retention such that it is similar to acyl chains 2 carbons shorter per each degree of unsaturation. For example, a C18:1 fatty acyl would have an elution order closer to a C16:0 fatty acyl; this is known as a critical pair (Gutnikov, 1995). This is true regardless of the head group moiety. Therefore, the methods described herein could be applied to any fatty acyl subclass with adjustment to the gradient elution program.

Normal phase separations of fatty acyls have been achieved with thin layer chromatography and adapted to solid phase extraction columns (Sultana and Johnson, 2006). These methods are capable of isolating cholesterol esters, fatty acids, monoacylglycerols, diacylglycerols, triacylglycerols, PFAMs, NAGs, and NAEs obtained from samples or sample extracts. Analyses of lipids have special process contamination and trace analysis aspects to consider. Several PFAMs and NAEs contaminants have been identified in different grades of solvents and as slip additives in plastics (Cooper and Tice, 1995; Skonberg et al., 2010). It is essential to test solvent background and to avoid plastics in all sample preparations. This usually requires the manual packing of solid phase extraction (SPE) columns, since commercial SPE cartridges are typically housed in plastic jackets. Additionally, lipids can adhere to glass surfaces; therefore, silinization of all glassware may be necessary to limit adherence and increase recovery of the analytes and/or increase their limit of detection.

Silver ion, or argenation, chromatography has been used extensively for separation of lipid samples with a high variation of unsaturation numbers and geometrical configuration, such as are commonly found in triacylglycerols (Dobson et al., 1995; Christie, 1998a,b; Momchilova et al., 1998; Nikolova-Damyanova et al., 2003; Adlof, 2004, 2007; Dugo et al., 2004, 2006a,b; Mondello et al., 2005; Christie et al., 2007). The general mechanism of action in this separation scheme is unclear, though Christie and co-workers propose a theory of silver ions forming weak, reversible charge transfer complexes with the analytes (Dobson et al., 1995; Christie, 1998c; Momchilova and Nikolova-Damyanova, 2003; Nikolova-Damyanova, 2009). Another possibility is an intermolecular ion-dipole interaction between the immobilized silver ions on the chromatographic bed and the π bonds of the analyte. In general, elution order of analytes by this technique is saturated, trans-unsaturated, mono-unsaturated, and so on. A number of experimental parameters and analyte variables have been explored in relation to retention factor allowing implementation of this methodology in a predictable manner (Christie, 1998b; Shan and Wilson, 2002; Harfmann et al., 2008). One exception to typical chromatographic separations is that retention of analytes in silver ion chromatography is reduced at lower temperature (Adlof and List, 2004).

Multidimensional liquid chromatography (MDLC) separations have been demonstrated for triacylglycerols in plant and animal samples (Dugo et al., 2004, 2006a,b; Mondello et al., 2005). Each approach utilized a reversed phase separation with an isopropanol/acetonitrile gradient and an isocratic silver ion separation with 0.5–0.7% acetonitrile in hexane. Samples that were separated with an inline MDLC approach had a reversed phase 2nd dimension separation with a total analysis time of less than 2 min (Mondello et al., 2005; Dugo et al., 2006a). More recently, the development of hybrid mass spectrometers with front-end mobility cells for ion mobility spectrometry capabilities has provided another powerful analytical tool for lipidomic studies that may be combined with chromatographic separations to effect 3D separations (Paglia et al., 2015).

To our knowledge, MDLC methods have not yet been published for PFAMs or NAGs, although our laboratory has utilized an SPE method prior to GC-MS analyses (Sultana and Johnson, 2006). In this report we provide effective separation methods developed for the bioactive PFAM and NAG lipid standards. Separations were effected by normal phase chromatography to differentiate lipid classes, and the peaks corresponding to PFAMs and NAGs were then further separated into their component species by reversed phase chromatography. PFAMs and NAGs were then characterized by MS/MS using either atmospheric pressure chemical ionization (APCI) or electrospray ionization (ESI), respectively. Given our laboratories interest in the effects of PFAMs on serotonergic systems, we then show the effectiveness of these general methods to isolate and identify PFAMs in brain tissue.

## Materials and methods

### Chemicals

Methanol (Optima grade), formic acid (Optima grade), ammonium acetate, hexane, acetonitrile, and ammonium hydroxide were purchased from Fisher Scientific (Fair Lawn, NJ, USA). Oxalyl chloride, oleic acid, erucic acid, petroselaidic acid, heptane HPLC grade, methyl-tert-butyl-ether HPLC grade, isopropanol HPLC grade, acetic acid, and anhydrous dichloromethane were from Sigma Aldrich (St Louis, MO, USA). N, N-dimethylformamide, heptadecanoic acid, and eicosanoic acid were purchased from Aldrich Chemical Company (Milwaukee, WI, USA). Lauric acid, myristic acid, palmitic acid, stearic acid, and docosanoic acid were purchased from Acros Organics (New Jersey, USA). Elaidic acid was from MP Biomedical Inc. (Solon, OH, USA) and linoleamide was purchased from Enzo Life Sciences (Ann Arbor, MI, USA). Stearoyl ethanolamine, oleoylglycine, linoleoylglycine, palmitoylglycine, arachidonoylglycine, and arachidoylglycine were purchased from Cayman Chemicals (Ann Arbor, MI, USA). 1 monopalmitoyl-rac-glycerol (MAG) and tristearin (TAG) were from Sigma (St. Louis, MO) 99% purity and 1,2-dipalmitoyl-rac-glycerol (DAG) was from MP Biomedicals (Solon, Ohio).

### Normal phase separation

PFAM and NAG standards were prepared in a mixture at 1 mM concentration of each standard. The mixture was separated via normal phase chromatography utilizing a YMC PVA-Sil column (4.6 × 250 mm, 5 μm particle size). Gradient elution is carried out starting at 95% mobile phase A (heptane with 0.5% v/v methyl-tert-butyl-ether) and increasing linearly to 50% mobile phase B (methyl-tert-butyl-ether with 10% v/v 2-propanol and 0.2% v/v acetic acid) over 40 min with a flow rate of 1 mL/minute. Fractions were collected at 1 min intervals with an injection volume of 200 μL and the times corresponding to NAG and PFAM elution were determined by reversed phase chromatography and MS/MS detection.

### Reversed phase separation of N-acyl glycines

Palmitoylglycine, linoleoylglycine, oleoylglycine, stearidonoylglycine, arachidonoylglycine, and arachidoylglycine were analyzed on an Agilent Technologies 1200 Liquid Chromatography system with a 6460 Triple Quadrupole Mass Spectrometry Detector. Mobile phase A was methanol and mobile phase B water, with 10 mM ammonium acetate in both phases. Separations were carried out on a YMC Cartenoid column (4.6 × 150 mm, 5 μm particle size) with a linear gradient of 90 to 100% mobile phase A over 15 min with a 15 min hold time. An additional separation method was developed on a Phenomenex C18 column (4.6 × 100 mm, 2.6 μm particle size) with a linear gradient of 80 to 100% mobile phase A over 5 min and a 2 min hold time. A second gradient method was used and consisted of 70% mobile phase A hold for 7 min, a linear gradient increase to 80% for 7 min, a 1 min hold at 80% before increasing to 100% mobile phase A over 5 min, and a final 5 min hold for a total analysis time of 25 min.

Ionization was achieved with an ESI source operated in negative mode with optimized parameters: fragmentor voltage 135 V, sheath gas flow 11 L/minute, nebulizer pressure 55 psi, nozzle voltage 500 V, capillary voltage 3500 V, drying gas flow 9 L/minute, drying gas temperature 275°C, and dwell time of 500 ms. Multiple reaction monitoring parameters were set-up to analyze the [M–H]^−^ parent ions and 74 *m/z* product ion representing the glycine head group fragment.

### Reversed phase separation of primary fatty acid amides

All primary fatty acid amide standards were synthesized in house at greater than 98% purity. Briefly, PFAMs were synthesized by a modified procedure described by Philbrook (1954) Fatty acids were dissolved in anhydrous dichloromethane and converted to the acid chloride by reaction with oxalyl chloride under anhydrous argon atmosphere. Dichloromethane solvent was removed by rotary evaporation *in vacuo*, and the remaining acid chloride was subjected to ammonia gas by inserting ammonium hydroxide filled syringes into the sealed, argon filled reaction vessel. Reactions were considered to be complete when the fatty acid chloride oil was completely converted to a white solid fatty acid amide product by visual inspection. Products were purified by liquid phase extraction with chloroform.

Purity of all synthesized amides was verified by GCMS on a Varian CP-3800 GC with Varian Saturn 2000 Ion Trap Mass Spectrometer. Gas chromatography was performed on a Varian Factor Four Capillary Column (VF-5 ms, 30 m × 0.25 mm ID) with a flow of 1 mL/min helium carrier gas. Injector temperature was held at 250°C with split injection (ratio 10). Temperature gradient started at 55°C and ramped 40°C/min to 150°C with a hold of 3.62 min before ramping 10°C/min to 275°C and holding 6.50 min. The total run time was 25 min. Eluted fatty amides were ionized by chemical ionization with methanol and analyzed in selected ion mode. The peak area of fatty acid substrate and PFAM product from GC-MS runs were used to determine purity. All PFAMs were found to be of 98% purity or greater. Lauramide (C12:0), myristamide (C14:0), linoleamide (C18:2^9,12^), palmitamide (C16:0), oleamide (C18:1^9^), elaidamide (C18:1^9trans^), petroselaidamide (C18:1^6trans^), heptadecanoamide (C17:0), stearamide (C18:0), arachidamide (C20:0), erucamide (C22:1^13^), and behenamide (C22:0) were separated on a Agilent RP C18 column (2.0 × 50 mm, 1.8 μm particle size) with a gradient elution of methanol and water, both containing 0.3% formic acid.

PFAMs were detected using an Agilent 6460 Triple Quadrupole Mass Spectrometer equipped with an atmospheric pressure chemical ionization (APCI) source. Optimized detection parameters are as follows: gas temperature 325°C, vaporization temperature 325°C, gas flow 4 L/minute, nebulizer pressure 22 psi, capillary voltage 3500 V, corona 4 μA, and fragmentor 125 V. Multiple reaction monitoring was used to detected the [M+H]^+^ parent ions and product ions of 55 and 43 *m/z* representing a short acyl chain and short alkene fragments, as determined from fragmentation studies for the monounsaturated and saturated compounds, respectively.

### Extraction of primary fatty acid amides from biological samples

Extraction of polar lipids from biological samples was achieved by a modified Folch-Pi method (Folch et al., 1957). All samples were extracted in glassware thoroughly cleaned by soap and water, distiller water rinse, 1 M sodium hydroxide solution soak for 1 h, distilled water rinse, acetone rinse, and toluene rinse. The dried glassware was silanized with trimethylchlorosilane (Seed, 2001). All plastic materials, including pipet tips, were avoided during all steps of analysis as PFAMs are common slip additives in plastic production (Cooper and Tice, 1995).

Samples of mouse brain (a generous gift from Dr. S. Amara, Univ. of Pittsburgh School of Medicine) were weighed and frozen at 20°C. The tissue sample was homogenized in a 2:1 chloroform:methanol solvent mixture containing 1 mM indomethancin with a volume of 20 times the sample weight. Heptadecanoamide was added as an internal standard to a final concentration of 10 μM. The insoluble material was removed by centrifugation and the supernatant was vortexed with an aqueous 10% KCl solution to remove salts, proteins, and water soluble components. The organic phase was dried under a stream of nitrogen and further separated by the normal phase separation method outlined in a previous section.

## Results

### Normal phase separation

**Figure 1:**
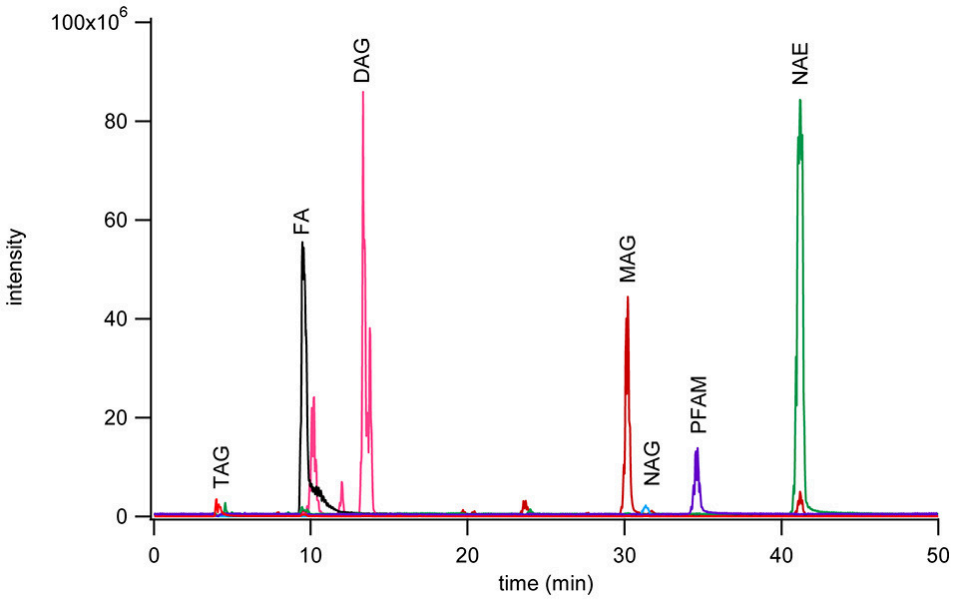
**Chromatogram of seven fatty acyl subclasses separated by normal phase chromatography using a YMC PVA-Sil (4.6 × 250 mm, 5 μm particle size) on a Waters ZMD MS with an ESI probe with polarity switching**. Separation was achieved with mobile phase A (heptane with 0.5% methyl-tert-butyl ether) and mobile phase B (10% 2-propanol, 0.2% acetic acid in methyl-tert-butyl ether) run in gradient mode from 95 to 50% A over 40 min. The monoacylglycerols were monitored as the [M+Na]^+^ peak, PFAM as the [M+H]^+^ peak, diacylglycerols as [M+Na]^+^ peak, NAE as [M+Na]^+^ peak, fatty acids as [M−H]^−^ peak, NAG as [M−H]^−^ peak, and triacylglycerols as [M+Na]^+^ peak. Each class was monitored on a different channel on the MS.

A 975 nmol lipid mixture of heptanoamide (FAs), tristearin (TAGs), 1,2-dipalmitoyl-rac-glycerol (DAGs), 1-monopalmitoyl-rac-glycerol (MAGs), N-oleoylglycine (NAGs), palmitamide (PFAMs), and stearoyl ethanolamine (NAEs) single representative standards from each lipid class were separated by normal phase chromatography (Figure 1). Sample injection volume was increased from 20 to 200 μL to accommodate larger scale sample purification needs. The effect of the increased injection volume on elution was tested by collecting one fraction per minute over the total gradient elution program. These fractions were dried down, reconstituted in methanol, and analyzed by reversed phase methods to check the elution range of the desired subclasses. The NAGs and PFAMs between C12 and C22 were found to co-elute from 31 to 38 min. The retention of these lipid classes deviate from that represented in Figure 1 due to a larger number of individual components in each class, vs. a single representative, and the increase in injection volume causing band broadening. Co-elution was determined not to be problematic because these species ionize in different modes for reversed phase MRM analysis.

### Reversed phase separation of N-acyl glycines

**Figure 2:**
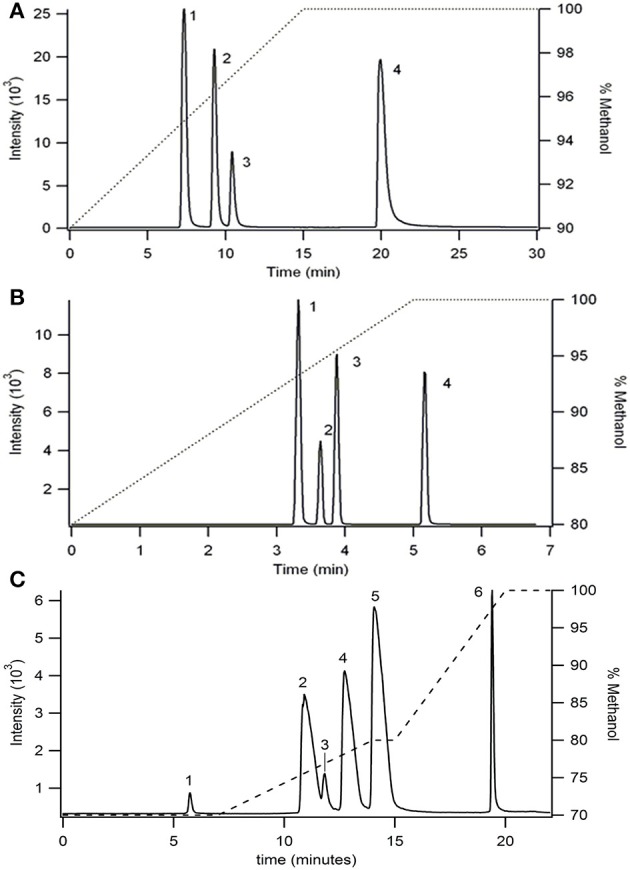
**Separation of NAGs on (A)** a C30 YMC carotenoid column (4.6 × 150 mm, 5 um particles size) and **(B,C)** Phenomenex C18 column (4.6 × 100 mm, 2.6 μm particles size) using two different gradient separations. Ionized by ESI and detected in MRM mode as the [M−H]^−^ parent and 74 *m/z* glycine head group product at collision energy of 20 V. Flow rate was 1 mL/min. The right axis shows the gradient elution profile for % methanol. Peak identities in **(A,B)** are (1) linoleoylglycine C18:2, (2) palmitoylglycine C16:0, (3) oleoylglycine C18:1, (4) arichidoylglycine C20:0, and **(C)** are (1) stearidonoylglycine C18:4, (2) linoleoylglycine C18:2, (3) arachidonoylglycine C20:4, (4) palmitoylglycine C16:0, (5) oleoylglycine C18:1, (6) arachidoylglycine C20:0.

Palmitoylglycine (C16:0), oleoylglycine (C18:1^9^), linoleoylglycine (C18:2^9,12^), stearidonoyolglyicne (C18:4^6,9,12,15^), arachidonoylglycine (C20:4^5,8,11,14^), and arachidoylglycine (C20:0) were separated utilizing a C30 YMC carotenoid column and a fused-core Phenomenex C18 column. Separation of palmitoylglycine (C16:0), oleoylglycine (C18:1^9^), linoleoylglycine (C18:2^9,12^), and arachidoylglycine (C20:0) was achieved on a C30 YMC carotenoid column (4.6 × 150 mm, 5 μm particles size) with gradient elution of methanol and water. Both mobile phases were modified with 10 mM ammonium acetate to aid in ionization. Elution was achieved by linear increase in methanol from 90 to 100% over 15 min, followed by a 15 min hold. Elution of each component was determined by identification of the parent mass ion (Figure 2A).

In an effort to reduce the analysis time, a fused-core Phenomenex Kinetix C18 column (4.6 × 100 mm, 2.6 μm particles size) was employed using methanol/water gradient elution (Figure 2B). The use of a fused-core particle C18 column reduced the experimental time by 6 fold compared to the C30 column separation while simultaneously increasing resolution.

A second elution method was developed (Figure 2C) using the fused-core C18 column to determine elution of two additional analytes, arachidonoylglycine (C20:4^5,8,11,14^) and stearidonoylglycine (C18: 4^6,9,12,15^). Although the total analysis time increased 4-fold compared with the previous gradient method (Figure 2B), co-elution between linoleoylglycine (C18:2) and arachidonoylglycine (C20:4) was reduced.

Utilizing the method developed in Figure 2C, the ionization parameters were optimized to yield the lowest detection limit possible. The limit of detection for each analyte was determined by 5 μL injection of standards between 100 nM and 10 μM. The results for limit of detection (LOD = signal to noise ratio, S/N, 5) and limit of quantitation (LOQ = S/N 10) are shown in Table 1. The calibration curves consisted of 5 points and were linear (*R*^2^ ≥ 0.994) between 1 and 10 μM.

**Table 1:** **LOD and LOQ for several commercially available NAGs determined by injection of 5 μL standard solutions from 100 nM to 10 μM**.

**N-acyl glycine**	**Abbreviation**	**LOD (μM)**	**LOQ (μM)**	**R^2^**
Stearidonoylglycine	C18:4^6,9,12,15^	0.475	1.00	0.999
Linoleoylglycine	C18:2^9,12^	0.499	1.00	0.999
Arachidonoylglycine	C20:4^5,8,11,14^	0.484	1.00	0.999
Palmitoylglycine	C16:0	1.00	1.00	0.994
Oleoylglycine	C18:2^9^	1.00	1.00	0.999
Arachidoylglycine	C20:0	1.00	1.00	0.999

*Calibration curves were linear between 1 and 10 μM. The LOD and LOQ were determined by a S/N of 5 and 10, respectively*.

### Reversed phase separation of primary fatty acid amides

**Figure 3:**
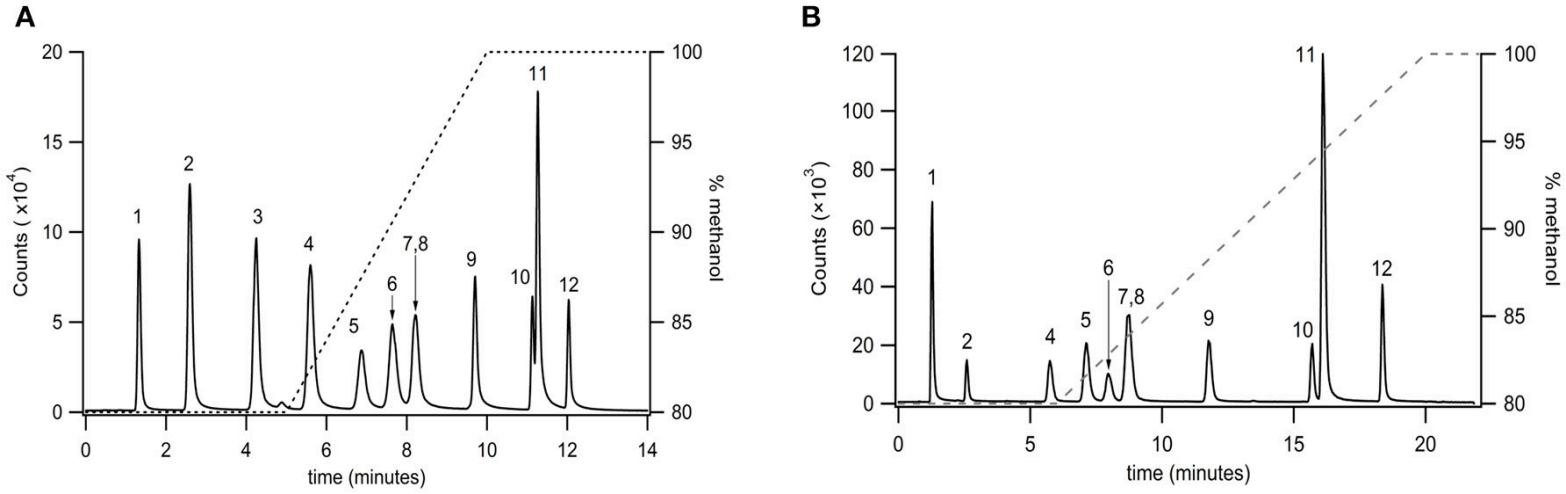
**Separation of very long chain PFAMs with Agilent RP C18 column (2.1 × 50 mm, 1.8 μm particle size) using a (A)** steep and **(B)** shallow gradient (% methanol on the right axis). Peak identities were identified by MS/MS, with fractions ionized by APCI and identified in MRM mode. The peaks were identified as (1) lauramide, (2) myristamide, (3) linoleamide, (4) palmitamide, (5) oleamide, (6) elaidamide, (7) petroselaidamide, (8) heptadecanoamide, (9) stearamide, (10) erucamide, (11) arachidamide, (12) behenamide.

Very long chain PFAMs (C12 to C22) were separated via reversed phase chromatography employing a sub 2 μm particle size column (Agilent RP C18 2.1 × 50 mm, 1.8 μm particle size). A gradient elution was established for a select group of PFAMs (Figure 3A), using a methanol/water gradient elution. All components tested were well resolved with the exception of erucamide (C22:1^13^) and arachidamide (C20:0). Extension of the linear gradient of 80–100% methanol from 5 to 10 min (Figure 3A) to 6–20 min (Figure 3B) resulted in baseline resolution of erucamide (C22:1^13^) and arachidamide (C20:0). Due to contamination of samples, as well as blanks and controls, with erucamide, the gradient separation in Figure 3A was used for separation of biological samples and erucamide was excluded from the MRM detection method.

Utilizing the separation method developed in Figure 3A, the optimized ionization parameters for detection were determined. The LOD for each analyte was determined by 2 μL injection of standards between 500 pM and 10 μM. The results for limit of detection (LOD = S/N, 5) and limit of quantitation (LOQ = S/N 10) are shown in Table 2. The calibration curves consisted of 5 points and were linear (*R*^2^ ≥ 0.997) between 0.500 and 10 μM on average. In cases where the LOD and LOQ were equal, the signal was lost below the LOD, however, at this concentration the S/N was above 10.

**Table 2:** **LOD and LOQ for select PFAMs determined by injection of 2 μL standard solutions from 500 pM to 10 μM**.

**PFAM**	**Abbreviation**	**LOD (nM)**	**LOQ (nM)**	**R^2^**
Lauramide	C12:0	50	100	0.999
Myristamide	C14:0	50	50	0.999
Linoleamide	C18:2^9,12^	10	50	0.999
Palmitamide	C16:0	20	50	0.999
Oleamide	C18:1^9^	400	400	0.999
Elaidamide	C18:1^9trans^	40	400	0.998
Petroselaidamide	C18:1^6trans^	40	400	0.999
Stearamide	C18:0	50	50	0.997
Erucamide	C22:1^13^	50	100	0.998
Arachidamide	C20:0	10	50	0.999
behenamide	C22:0	20	20	0.999

*Calibration curves were linear between 0.500 and 10 μM. The LOD and LOQ were determined by a S/N of 5 and 10, respectively*.

### Separation and detection of primary fatty acid amides in biological samples

**Figure 4:**
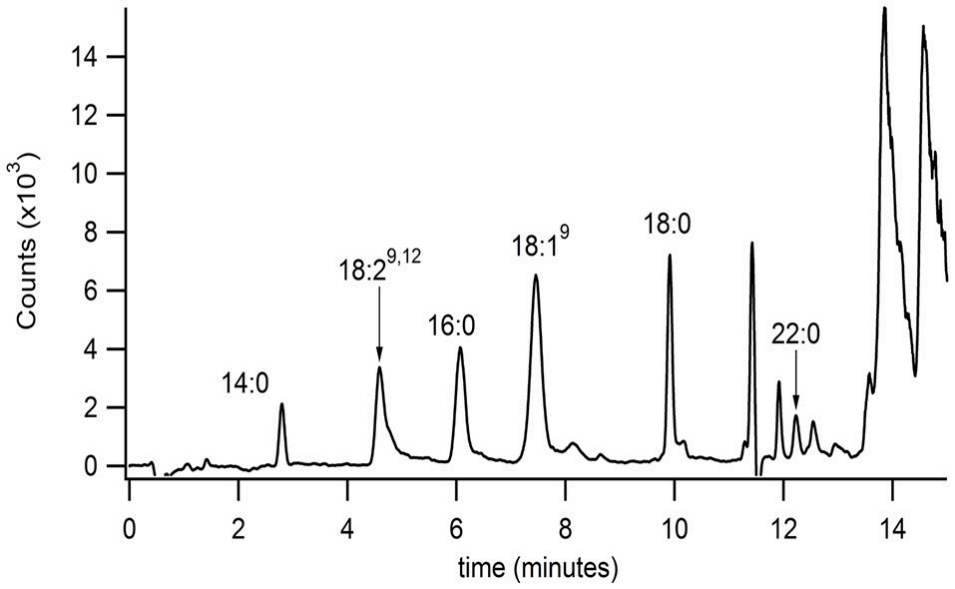
**Isolation and identification of PFAMs in brain**. The equivalent of 10 Swiss-Webster mouse brains (with mid brain removed) were Folch-Pi extracted. Samples were then subjected to MDLC using normal phase chromatography, and the peak corresponding to PFAM class of lipids were then separated by reversed phase chromatographs (as described in the text). PFAM substituents where identified by MS/MS as described Figure 3. PFAM substituents corresponding to 14:0, 19:2^9,12^, 16:0, 18:1^9^, 18:0, and 22:0 PFAMs were identified in mouse brain tissue by MRM (as described in the text).

The developed extraction, normal phase, and reversed phase separation methods were used serially to analyze PFAM content in a sample of mouse brain (Figure 4). These studies were conducted to validate the described methodology and ensure the correct method parameters at each of the 3 separate steps, extraction, normal phase lipid class selection, and reversed phase separation and detection. In addition the validation aimed to determine the sensitivity of the method in a complex matrix. Initial attempts failed to reproducibility detect lipids in tissue samples. Several factors in the extraction process were identified which contributed to reproducibility. Plastic materials were avoided due to leachable PFAM contamination (Cooper and Tice, 1995). The samples were frozen and indomethacin was added to the extraction solvent to reduce the rate of PFAM catabolism.

Brain tissue was chosen due to the documented interaction of oleamide and neuroreceptors, such as 5HTR. Myristamide (C14:0), linoleamide (C18:2), palmitamide (C16:0), oleamide (C18:1), stearamide (C18:0), and behenamide (C22:0) were identified in mouse brain tissue extracts by matching retention time and parent ion mass. An internal standard, heptadecanoamide, which was added prior to homogenization of the tissue samples, was recovered at 72% confirming collection of the correct fraction in the normal phase separation. The blank sample had a similar % recovery of the internal standard with no PFAM peaks present (data not shown).

## Discussion and conclusion

This work describes various strategies for efficient separation and detection of saturated and unsaturated fatty acyls. Different subclasses of lipids were resolved with a normal phase separation scheme utilizing a heptane and methyl-tert-butyl ether mobile phase and gradient elution. The addition of isopropanol to the mobile phase was necessary to increase the solubility of these lipids, reducing the carry over between injections. Nonetheless, it was still essential to occasionally wash the column with polar solvents, especially if large injection volumes (>100 μL) were used frequently. The fatty acyl subclasses separated via the normal phase method are those commonly extracted with Folch-Pi from biological samples. This method is comparable to previously reported SPE methods (Sultana and Johnson, 2006) and although it has an increased total analysis time the separation is automated and reproducible. The eluent can be monitored by MS if a post-column feed is used or collected directly for further separation and analyses. It was found that, with a 200 μL injection volume, the PFAMs and NAGs co-elute as a single peak between 31 and 38 min. This, however, was not problematic as subsequent MS/MS studies found that NAGs preferentially ionized in the negative mode while PFAMs were observed in positive mode, allowing easy discrimination between the co-eluting lipids.

Following separation of fatty acyls with normal phase the individual subclasses (e.g., PFAMs or NAGs) can be further separated to determine the distinct analytes present. This was achieved with C18 reversed phase chromatography and detected with tandem MS. The elution order followed the trends observed with fatty acids eluted from reversed phase columns (Gutnikov, 1995). The “critical pairs” were separated by adjusting the gradient elution parameters and/or increasing the column theoretical plates. For PFAMs, isobaric compounds (oleamide C18:1^9^, elaidamide C18:1^9trans^, and petroselaidamide C18:1^6trans^) differing only in double bond position were resolved using a 5 min hold at 80% methanol followed by a 5 min linear ramp to 100% methanol. This resulted in LOD of 10–400 nM was obtained depending on the species. NAGs proved to be difficult to separate on standard C18 columns due to low solubility and increased interaction with the stationary phase. Therefore, a C30 substituted column and a fused-core C18 column with a reduced particle size were employed to determine the optimum conditions for NAG separation. Of note, in our hands NAGs were not found to ionize efficiently or reproducibly in positive ion mode, thus, negative ion mode was used for all NAG studies. The C30 column proved useful in separation of saturated and monounsaturated NAGs with modest tailing, however, the analysis time was undesirable when considering MDLC. The 2.6 μm particle size fused-core column was expected to reduce the analysis time, peak broadening, and the tailing factor. As expected, the analysis time was reduced by 6-fold and peaks were baseline resolved. Due to the low ionization efficiency of these compounds, the LOD was 1 μM for all species, consequently limiting the ability to detect physiological levels.

Utilizing the developed methodology, PFAMs were successfully detected in extracts of mouse brain tissue. PFAMs and NAEs have been reported in biological tissues at pmol/g of tissue. Though the utilized methodology has successfully identified PFAMs in brain tissue, the levels are not sufficient for quantitation. It is clear, even with careful method selection and sample processing consideration, that quantitation of trace levels of the PFAM and NAG subclasses pose a challenge for conventional separation and detection methods. Several factors were found to have a profound impact on analysis. When selectively analyzing for fatty amides the use of all plastic containing equipment and sample vessels should be eliminated. Under Folch-Pi extraction conditions, these substrates leach fatty amides, thus, contaminating the sample matrix (Cooper and Tice, 1995).

Additionally, rigorous care should be taken to fully control experimental conditions before and after tissue excision. Quantitation studies on fatty acid ethanolamines in biological samples have found similar effects with tissue quality (Epps et al., 1979; Skaper et al., 1996; Giuffrida and Piomelli, 1998; Kondo et al., 1998). In our study, addition of indomethacin to the 2:1 chloroform/methanol solvent had a positive effect on detection of components in extracted tissue samples as early extraction attempts without indomethacin did not detect PFAMs in tissue samples. Extra steps were taken to perform extractions when tissues were frozen with the addition of indomethacin to the extraction solution. After these method adjustments, PFAMs were detected in the tissue samples. A cocktail of inhibitors with the use of a trapping MS may provide the sensitivity needed to overcome the LOQ.

In summary, we developed an off-line MDLC system for analysis of PFAMs and NAGs. Using lipid standards, normal phase separation was capable of automated separation of complex biological lipid matrices with comparable sample recovery to SPE. Each individual subclass of lipids could be successfully sampled from the normal phase and further resolved into each individual component with a secondary reversed phase method. Fused core particle and sub-2 μm column packings enabled high resolution separation of lipid lengths from C12 to C22 within 12 min. This included resolution of several positional isomers of C18:1, some of which have been shown to affect physiological states in vertebrates. The MDLC coupled with MS/MS was then shown to be capable in identifying PFAMs in samples with a more complex lipid composition, homogenized mouse brain. The methodology described herein provides a framework for future analyses aimed at identifying and elucidating the roles and significance of lipids in health and disease.

## Author contributions

All experimental studies were conducted by ED, except for the initial normal phase chromatography trials that were conducted by KK. Studies were directed by MC and initial drafts were written by ED and MC.

### Conflict of interest statement

The authors declare that the research was conducted in the absence of any commercial or financial relationships that could be construed as a potential conflict of interest.
